# Maximizing plyometric training for adolescents: a meta-analysis of ground contact frequency and overall intervention time on jumping ability: a systematic review and meta-analysis

**DOI:** 10.1038/s41598-023-48274-3

**Published:** 2023-12-01

**Authors:** Lunxin Chen, Zijing Huang, Lin Xie, Jiaxin He, Hongshen Ji, Weifeng Huang, Duanying Li, Yanfeng Zhou, Jian Sun

**Affiliations:** 1 Digitalized Strength and Conditioning Training Laboratory, Guangzhou Sport University, Guangzhou, China; 2 School of Athletic Training, Guangzhou Sport University, Guangzhou, China

**Keywords:** Paediatric research, Quality of life

## Abstract

Plyometric training boosts adolescents' jumping ability, crucial for athletic success and health. However, the best total ground contact frequency (TGCF) and overall intervention time (OIT) for these exercises remain unclear. This meta-analysis aims to identify optimal TGCF and OIT in plyometric training for adolescents, focusing on countermovement jump (CMJ) and squat jump (SJ) outcomes. This systematic review encompassed five databases and included 38 studies with 50 randomized controlled experiments and 3347 participants. We used the Cochrane risk assessment tool for study quality and Review Manager 5.4 for data analysis. The current meta-analysis incorporated a total of 38 studies, comprising 50 sets of randomized controlled trials, to investigate the influence of different TGCFs and OITs on plyometric training. The Cochrane risk assessment tool indicated that all the included studies were classified as low risk. Various TGCFs in plyometric training positively affected CMJ and SJ heights in adolescents. The TGCF of less than 900 was ideal for enhancing CMJ, whereas more than 1400 was effective for SJ. The optimal OIT was 400–600 min, specifically, 500–600 min for CMJ and 400–500 min for SJ. Plyometric training improves jumping ability in adolescents. Lower ground contact frequency (< 900 contacts) enhances CMJ, while higher ground contact frequency (> 1400 contacts) is more effective for SJ. Optimal intervention time ranges from 400 to 600 min, with 500 to 600 min benefiting CMJ and 400 to 500 min improving SJ.

## Introduction

Jumping ability is a fundamental physical skill that is of great importance to the development of adolescents^1,2^. In many sports such as basketball, volleyball, high jump, and long jump, good jumping ability is essential for young athletes and is closely related to their performance in these events^3^. For ordinary adolescents, good jumping ability is also important. Studies have shown that jumping ability is closely related to adolescents' level of physical activity and quality of life^4^. In addition, physical ability is one of the important factors contributing to adolescents' self-esteem and psychological health^5^. By participating in jump training, ordinary adolescents can improve their motor skills, enhance their self-confidence, promote physical health, and establish good exercise habits^6^.

Plyometric training is a commonly used method for physical conditioning^7^. It has been shown to effectively improve the jumping ability of adolescents^8–11^. This effectiveness is due to the effective utilization of both mechanical and neurophysiological models by the plyometric training. The mechanical model suggests that when a muscle is rapidly stretched, elastic potential energy is increased and stored^12,13^. When the stretched muscle is immediately shortened with a rapid concentric contraction, the stored elastic potential energy is instantly released, thereby increasing the muscle's force output^12,13^. In the neurophysiological model, the muscle utilizes the principle of the stretch reflex to enhance the force of the concentric contraction^14–17^. However, for adolescents, many variables may affect the effectiveness of plyometric training.

Total ground contact frequency (TGCF) and overall intervention time (OIT) appear to be two key variables that affect the effectiveness of plyometric training. TGCF refers to the number of ground contacts made by an athlete during a complete plyometric training period^18^. OIT refers to the total duration of plyometric training performed by an athlete during a training period^19^. Both of these variables are both crucial factors that impact training effectiveness and sport performance.^20^. This is attributed to the fact that higher TGCF and OIT generally indicate a greater training intensity, which can help improve muscle contraction speed and strength, thereby enhancing athletic performance^20^. However, there is currently insufficient evidence to determine the optimal range of TGCF for improving jumping ability in adolescents, and there is a lack of recommendations for the OIT of plyometric training for adolescents.

Given the importance of jumping ability in adolescents and the relationship between TGCF and OIT of plyometric training with training effectiveness and athletic performance, it is particularly important to explore the appropriate TGCF and reasonable OIT for adolescents undergoing plyometric training. Therefore, this systematic review with meta-analysis aimed to analyze the optimal values of total TGCT and OIT for enhancing jumping ability, specifically countermovement jump (CMJ) and squat jump (SJ) in adolescents. By addressing the current lack of specific guidance regarding TGCT and OIT, the present review endeavors to provide valuable recommendations and guidance in the field of plyometric training for adolescents.

## Information and research methods

The present systematic review and meta-analysis followed the PRISMA guidelines, and the protocol has been registered in PROSPERO (ID: CRD42023446372).

### Search strategy

**Figure 1:**
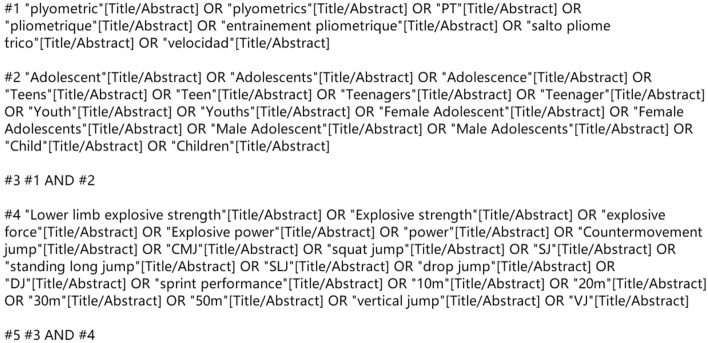
PubMed Literature Selection Strategy.

The present systematic review and meta-analysis conducted searches in databases including PubMed, Web of Science, Scopus, ProQuest, and China National Knowledge Infrastructure (CNKI). The search process is illustrated in Fig. 1 (PubMed was used as an example). The search time frame extended from the beginning of records in each database to February 6th, 2023. To ensure the accuracy of the search, two researchers (D.L and L.C) cross-checked the search keywords. If there were any disagreements on keyword selection between the two researchers, a third researcher (Z.H) would make the final decision. If necessary, manual searches would be conducted to supplement the literature.

### Study selection

The literature inclusion criteria for this meta-analysis were based on the participants, intervention, comparison, outcome, and study (PICOS) format of evidence-based medicine.

The inclusion criteria were as follows: (1) the study participants were adolescents aged 10–19 years^21^; (2) the experimental group underwent plyometric training as an intervention, followed by their participation in specialized training (such as football, basketball, volleyball, etc.) or regular physical education classes, which were identical to those provided to the control group. The control group did not receive the intervention of plyometric training but engaged in activities similar to the experimental group, including specialized training or regular physical education classes; (3) the control group served as a blank control, receiving no intervention of plyometric training, but participating solely in specialized training or regular physical education classes; (4) all studies were conducted on rigid ground to avoid interference from different training surfaces; (5) the outcome measures were jump performance indicators, including CMJ and SJ; (6) all studies should include one of the following variables: TGCF or OIT; (7) the study design was a randomized controlled trial (RCT).

The exclusion criteria were as follows: (1) non-randomized controlled trials, self-controlled trials, and randomized crossover trials; (2) inability to obtain study data; (3) conference papers, reviews, and comments; (4) unable to obtain full-text articles; (5) outside the age range of adolescents.

**Figure 2:**
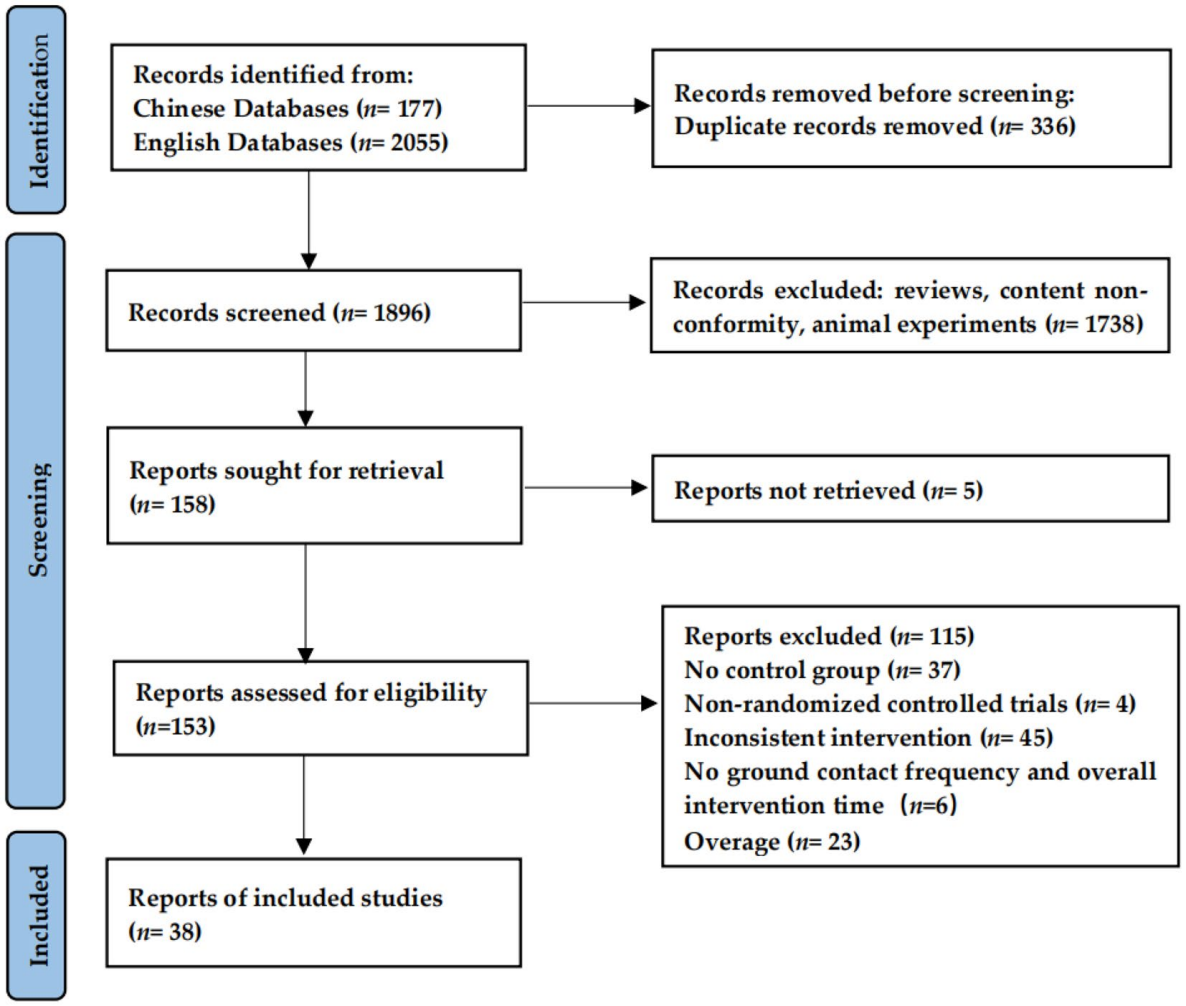
PRISMA flow chart for inclusion and exclusion of studies.

A total of 38 studies that met the inclusion criteria were included in the analysis. The detailed inclusion and exclusion process is shown in the figure below (Fig. 2).

### Data extraction

To avoid duplications, all literature from the various databases was imported into the EndnoteX9 software for deduplication. After confirming all the final literature included in the analysis, two researchers (C.L and H.Z) extracted the data into a Microsoft Excel spreadsheet. The extracted data included author’s name, publication year, characteristics of the subjects (such as age or maturation stage, gender, sample size), pre- and post-test data for the included indicators, training program details (such as TGCF, OIT, intervention period, training frequency, intervention methods for the experimental and control groups). If there were any discrepancies in the data extracted by the two researchers, a third researcher (Z.M) would extract and confirm the data. All pre- and post-test data were included in the analysis as mean ± standard deviation and were converted into change values ± standard deviation by one researcher (C.L) before being ultimately included in the analysis. If the full text or study data were unavailable, the corresponding author was contacted to obtain the relevant information.

### Assessment of risk of bias

The Cochrane risk of bias assessment tool was used to evaluate the quality of all included studies, and each criterion was assessed as "low risk of bias," "unknown risk of bias," or "high risk of bias." The studies were classified into A, B, or C quality levels based on the number of "low risk of bias" criteria met: A level for four or more, B level for two to three, and C level for one or none^8^. Two researchers (C.L and H.Z) independently assessed the quality of all studies included in this meta-analysis.

### Statistical analysis

The data analysis was conducted using the Review Manager 5.4 software (Review Manager, The Nordic Cochrane Centre, Copenhagen, Denmark). To ensure the objectivity and persuasiveness of the study, the present meta-analysis and subgroup analysis were conducted with data from no less than two groups^22^. All units of measurement were converted to a common unit (e.g., all units of length were converted to centimeters). Therefore, the weighted mean difference (WMD) with a 95% confidence interval was used as the overall effect size indicator in the final analysis. The heterogeneity index I^2^ was used to evaluate the heterogeneity of the studies. I^2^ values lower than 25%, between 25 and 75%, and higher than 75% are considered to have negligible, moderate, and high heterogeneity, respectively^23^. If the heterogeneity of the studies was less than 25%, a fixed-effect model was used for outcome indicator analysis. If the heterogeneity was greater than 25%, a random-effect model was used for outcome indicator analysis^8^. *P* < 0.05 was considered statistically significant.

Since there are currently no studies that classify different TGCF, this study used an artificial classification method to present the research results more persuasively. The aim was to ensure that each subgroup contained a comparable number of studies, thereby increasing the comparability and persuasiveness among the subgroups. Specifically, different TGCFs were divided into three groups: low ground contact frequency (LGCF), medium ground contact frequency (MGCF), and high ground contact frequency (HGCF). LGCF included experiments with a TGCF of less than 900, MGCF included experiments with a TGCF between 900 and 1400, and HGCF included experiments with a TGCF greater than 1400. In addition, OIT was classified by every 100 min as a subgroup, for example, 200–300 min as a subgroup. This finer classification aimed to provide a more detailed analysis. If the number of articles in each subgroup was less than 2, the subgroup would be canceled and not included in the analysis.

### Ethics approval

The study has been registered in the International Prospective Register of Systematic Reviews (PROSPERO: CRD42023446372).

## Results

### Study characteristics

According to the PRISMA reporting guidelines, 38 studies comprising 50 experimental groups were included in this meta-analysis. The studies involved 3347 adolescent participants aged 10–19 years, including those with specialized training and those without. The intervention in the experimental group was a plyometric training, while the control group received only specialized training without any other training intervention. The majority of the studies had an intervention time of 20–40 min, an intervention period of 6–10 weeks, and an intervention frequency of twice a week (Table 1).

**Table 1:** Characteristics of study participants.

Studies	Genders	Sample size		Age		Experimental group						Control group
		PT	CON	PT	CON	Interventions	PT Duration	Frequency	Duration	TGCF	OIT	Interventions
Asadi 2018(PRE-PHV)^79^	Male	10	10	11.5 ± 0.8	11.7 ± 0.4	PT + Soccer	30–40 min	2 week	6 weeks	720	420	Soccer
Asadi 2018 (Mid-PHV)^79^	Male	10	10	14.0 ± 0.7	14.2 ± 0.6	PT + Soccer	30–40 min	2 week	6 weeks	720	420	Soccer
Asadi 2018 (POST-PHV)^79^	Male	10	10	16.6 ± 0.6	16.2 ± 0.3	PT + Soccer	30–40 min	2 week	6 weeks	720	420	Soccer
Attene 2015^80^	Female	18	18	14.83 ± 0.92	15.20 ± 0.92	PT + Basketball	20 min	2 week	6 weeks	1120	240	Basketball
Carlos 2022^81^	Male – Female	44	39	10.7 ± 0.4	10.8 ± 0.5	PT + PE	30 min	2 week	8 weeks	–	480	PE
Chelly 2014^82^	Male	12	11	17.1 ± 0.3	17.2 ± 0.4	PT + Handball	30 min	2 week	8 weeks	860	480	Handball
Chelly 2015^83^	Male	14	13	11.7 ± 1.0	12.1 ± 1.0	PT + Track and Field	20 min	3 week	10 weeks	–	600	Track and Field
de Villarreal 2021^84^	Male	10	10	13.57 ± 1.39	14.66 ± 0.86	PT + Basketball	20 min	2 week	7 weeks	1960	280	Basketball
Drouzas 2020^85^	Male	23	22	10.0 ± 0.5	10.2 ± 1.6	PT + Soccer	15 min	2 week	10 weeks	1440	300	Soccer
Du 2020^86^	Male – Female	13	13	17.31 ± 0.48	17.23 ± 0.44	PT + Latin dance	90 min	3 week	8 weeks	2096	720	Latin dance
Fathi 2018^87^	Male	20	20	14.6 ± 0.5	14.5 ± 0.6	PT + Volleyball	20 min	2 week	16 weeks	1184	640	Volleyball
Fischetti 2018^88^	Male	10	12	13.7 ± 0.5	13.5 ± 0.5	PT + Track and Field	90 min	2 week	8 weeks	–	240	Track and Field
Hall 2016^89^	Female	10	10	12.5 ± 1.67	12.5 ± 1.67	PT + Gymnastics	60 min	2 week	6 weeks	1114	720	Gymnastics
Hammami 2019^90^	Male	14	12	15.7 ± 0.2	15.8 ± 0.2	PT + Soccer	35 min	2 week	8 weeks	722	560	Soccer
Hernández 2018^91^	Male	7	6	10.0 ± 1.5	9.7 ± 2.0	PT + Basketball	40 min	2 week	7 weeks	–	560	Basketball
Idrizovic 2018^92^	Female	13	17	16.6 ± 0.6	16.6 ± 0.7	PT + Volleyball	20–30 min	2 week	12 weeks	1226	540	Volleyball
Ignacio 2021 (NPPT)^93^	Male	8	7	13.8 ± 2.0	14.1 ± 0.2	PT + Basketball	–	2 week	6 weeks	2304	–	Basketball
Ignacio 2021 (PPT)^93^	Male	7	7	14.6 ± 1.1	14.0 ± 2.0	PT + Basketball	–	2 week	6 weeks	2304	–	Basketball
Jlid 2019^94^	Male	14	14	11.8 ± 0.4	11.6 ± 0.5	PT + Soccer	20–25 min	2 week	8 weeks	1596	360	Soccer
Kosova 2022^95^	–	14	12	15.16 ± 0.74	15.22 ± 0.86	PT + Fencing	–	3 week	8 weeks	1980	–	Fencing
Lioyd 2016 (PRE-PHV)^2^	Male	10	10	12.7 ± 0.3	12.7 ± 0.3	PT + PE	–	2 week	6 weeks	958	–	PE
Lioyd2016(POST-PHV)^2^	Male	10	10	16.4 ± 0.2	16.2 ± 0.3	PT + PE	–	2 week	6 weeks	958	–	PE
Marta 2022^81^	Male – Female	41	39	10.84 ± 0.47	10.84 ± 0.47	PT + PE	30 min	2 week	8 weeks	–	480	PE
Marzouki 2022 (Boys)^96^	Male – Female	20	20	10.1 ± 1.2	10.0 ± 1.2	PT + PE	–	2 week	4 weeks	676	–	PE
Marzouki 2022 (Girls)^96^	Male – Female	20	20	10.0 ± 1.1	10.1 ± 1.1	PT + PE	–	2 week	4 weeks	676	–	PE
Meszler 2019^97^	Female	9	9	15.8 ± 1.2	15.7 ± 1.3	PT + Basketball	20 min	2 week	7 weeks	1027	280	Basketball
Michailidis 2013^98^	Male	24	21	10.6 ± 0.6	10.6 ± 0.5	PT + Soccer	20–25 min	2 week	12 weeks	–	660	Soccer
Negra 2020 (4 weeks)^64^	Male	11	11	12.7 ± 0.3	12.8 ± 0.3	PT + Soccer	35–40 min	2 week	4 weeks	–	300	Soccer
Negra 2020 (8 weeks)^64^	Male	11	11	12.7 ± 0.12	12.8 ± 0.12	PT + Soccer	35–40 min	2 week	8 weeks	–	600	Soccer
Negra 2020 (12 weeks)^64^	Male	11	11	12.7 ± 0.13	12.8 ± 0.13	PT + Soccer	35–40 min	2 week	12 weeks	–	900	Soccer
Nurper 2014^99^	Female	9	9	18.3土2.6	18.0土2.0	PT + Soccer	60 min	1 week	8 weeks	1210	480	Soccer
Padrón-Cabo 2021^100^	Male	10	10	12.60 ± 0.70	12.39 ± 0.56	PT + Soccer	20–35 min	2 week	6 weeks	512	270	Soccer
Potdevin 2011^101^	Male – Female	12	11	14.3 ± 0.2	14.1 ± 0.2	PT + Swimming	20–25 min	2 week	6 weeks	2146	270	Swimming
Ramirez-Campillo 2018(FIXED)^20^	Male	25	24	13.9 ± 1.9	13.7 ± 1.6	PT + Soccer	13 min	2 week	7 weeks	906	182	Soccer
Ramirez-Campillo 2018(OPT)^20^	Male	24	24	13.1 ± 1.7	13.7 ± 1.6	PT + Soccer	13 min	2 week	7 weeks	906	182	Soccer
Ramirez-Campillo 2019^102^	Male	19	20	13.2 ± 1.8	13.5 ± 1.9	PT + Soccer	20 min	2 week	7 weeks	840	280	Soccer
Ramirez-Campillo 2020^103^	Male	8	7	12.9 ± 1.9	12.6 ± 1.8	PT + Soccer	10–15 min	2 week	8 weeks	810	200	Soccer
Ramirez-Campillo 2020(After)^104^	Male	14	12	17.1 ± 0.3	17.1 ± 0.5	PT + Soccer	20 min	2 week	7 weeks	1404	280	Soccer
Ramirez-Campillo 2020(Before)^104^	Male	12	12	16.9 ± 0.7	17.1 ± 0.5	PT + Soccer	20 min	2 week	7 weeks	1404	280	Soccer
Romero 2021(7th-grade)^105^	Female	10	9	12.7 ± 0.6	12.8 ± 0.6	PT + PE	21 min	2 week	6 weeks	1590	252	PE
Romero 2021(10th-grade)^105^	Female	9	9	16.3 ± 0.5	16.2 ± 0.5	PT + PE	21 min	2 week	6 weeks	1590	252	PE
Sammoud 2019^106^	Male	14	12	10.3 ± 0.4	10.5 ± 0.4	PT + Swimming	25–30 min	2 week	8 weeks	1360	440	Swimming
Sammoud 2021^107^	Female	12	10	10.01 ± 0.57	10.50 ± 0.28	PT + Swimming	25–30 min	2 week	8 weeks	1360	440	Swimming
Sedano 2011^108^	–	11	11	18.4 ± 1.1	18.2 ± 0.9	PT + Soccer	–	3 week	10 weeks	2880	–	Soccer
Tottori 2019^109^	Male	11	15	10.3 ± 0.7	10.9 ± 0.9	PT + PE	60 min	1 week	8 weeks	795	480	PE
Uzelac-Sciran 2020 (PRE-PHV)^110^	Male	24	19	13.1 ± 0.6	13.2 ± 0.5	PT + PE	40 min	2 week	8 weeks	1336	640	PE
Uzelac-Sciran 2020 (POST-PHV)^110^	Male	33	8	14.0 ± 0.4	13.9 ± 0.5	PT + PE	40 min	2 week	8 weeks	1336	640	PE
Vera-Assaoka 2019(early)^7^	Male	16	16	11.2 ± 0.8	11.5 ± 0.9	PT + Soccer	21 min	2 week	7 weeks	840	294	Soccer
Vera-Assaoka 2019(late)^7^	Male	22	22	14.4 ± 1.0	14.5 ± 1.1	PT + Soccer	21 min	2 week	7 weeks	840	294	Soccer
Zribi 2014^111^	Male	25	26	12.1 ± 0.6	12.2 ± 0.4	PT + Basketball	15–20 min	2 week	9 weeks	–	315	Basketball

*PT* plyometric training, *CON* control group, *CMJ* countermovement jump, *SJ* squat jump, *TGCF* total ground contact frequency, *OIT* overall intervention time, *PE* physical education, min minute, *PPT* progressed plyometric training, *NPPT* non-progressed plyometric training, *FIXED* fixed drop-box height, *OPT* optimal drop-box height, *After* plyometric training applied after soccer practice, *Before* plyometric training applied before soccer practice, *early* tanner stage maturation of 1–3, *late* tanner stage maturation of 4–5.

### Risk of bias in the included articles

**Figure 3:**
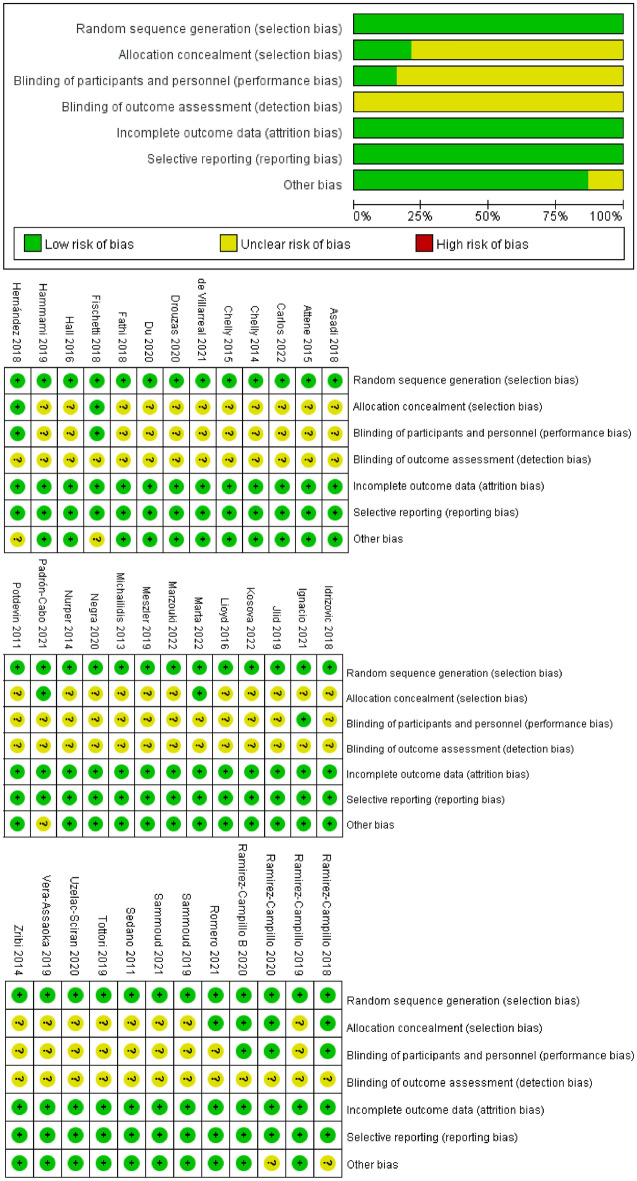
Judgments about each risk-of-bias item for each included study and risk-of-bias item presented as percentages across all included studies.

The Cochrane risk of bias assessment tool was used to assess the quality of all the literature included in this meta-analysis. All the studies included in this analysis were randomized controlled trials, with 8 studies reporting allocation concealment and 6 studies implementing blinding of the researchers and participants. All the studies included in this meta-analysis were rated as low risk of bias (Fig. 3).

### Meta-analysis results

#### The impact of plyometric training with varying ground contact frequencies on jumping ability in adolescents

**Figure 4:**
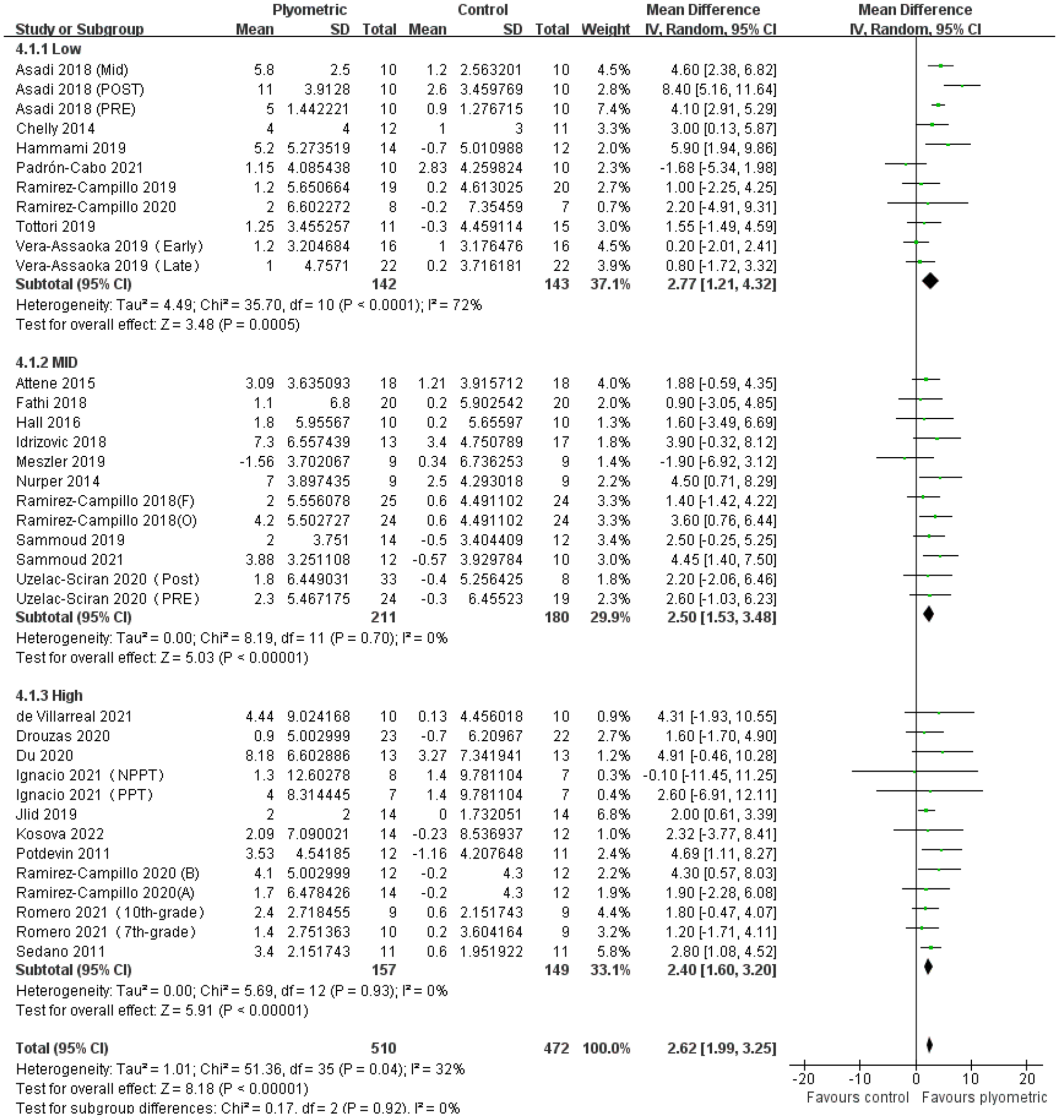
Forest plot of the effect of plyometric training with varying ground contact frequencies on CMJ height in adolescents.

A total of 28 studies comprising 36 experimental groups and 982 participants were included in this meta-analysis to evaluate the impact of plyometric training with varying ground contact frequencies on CMJ height in adolescents (Fig. 4). The overall effect size showed a significant positive effect of plyometric training on CMJ height in adolescents [MD = 2.62, 95% CI (1.99, 3.25)], with moderate heterogeneity (I^2^ = 32%) and statistical significance (*P* < 0.01). Subgroup analysis indicated that plyometric training with different TGCFs had a positive effect on CMJ height in adolescents, including LGCF [MD = 2.77, 95% CI (1.21, 4.32), *P* < 0.01, I^2^ = 72%], MGCF [MD = 2.50, 95% CI (1.53, 3.48), *P* < 0.01, I2 = 0%’], and HGCF [MD = 2.40, 95% CI (1.60, 3.20), *P* < 0.01, I^2^ = 0%].

**Figure 5:**
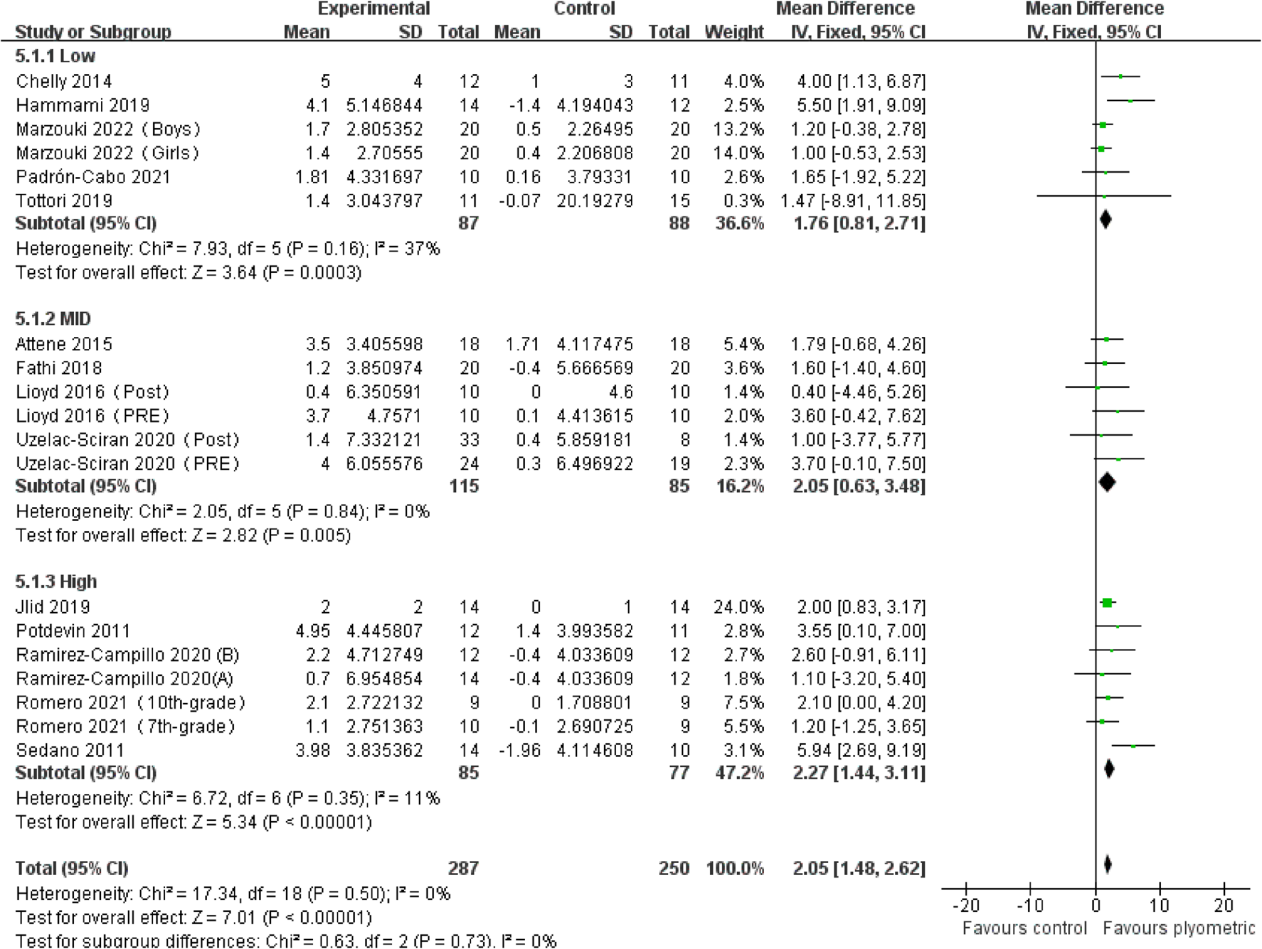
Forest plot of the effect of plyometric training with varying ground contact frequencies on SJ height in adolescents.

A total of 14 studies comprising 19 experimental groups and 537 participants were included in this meta-analysis to evaluate the impact of plyometric training with varying ground contact frequencies on SJ height in adolescents (Fig. 5). The overall effect size showed a significant positive effect of plyometric training on SJ height in adolescents [MD = 2.05, 95% CI (1.48, 2.62)], with no heterogeneity among the studies (I^2^ = 0%) and statistical significance (*P* < 0.01). Subgroup analysis indicated that plyometric training with different TGCFs had a positive effect on SJ height in adolescents, including LGCF [MD = 1.76, 95% CI (0.81, 2.71), *P* < 0.01, I^2^ = 37%], MGCF [MD = 2.05, 95% CI (0.63, 3.48), *P* < 0.01, I^2^ = 0%], and HGCF [MD = 2.27, 95% CI (1.44, 3.11), *P* < 0.01, I^2^ = 11%].

#### The impact of plyometric training with different OITs on jumping ability in adolescents

**Figure 6:**
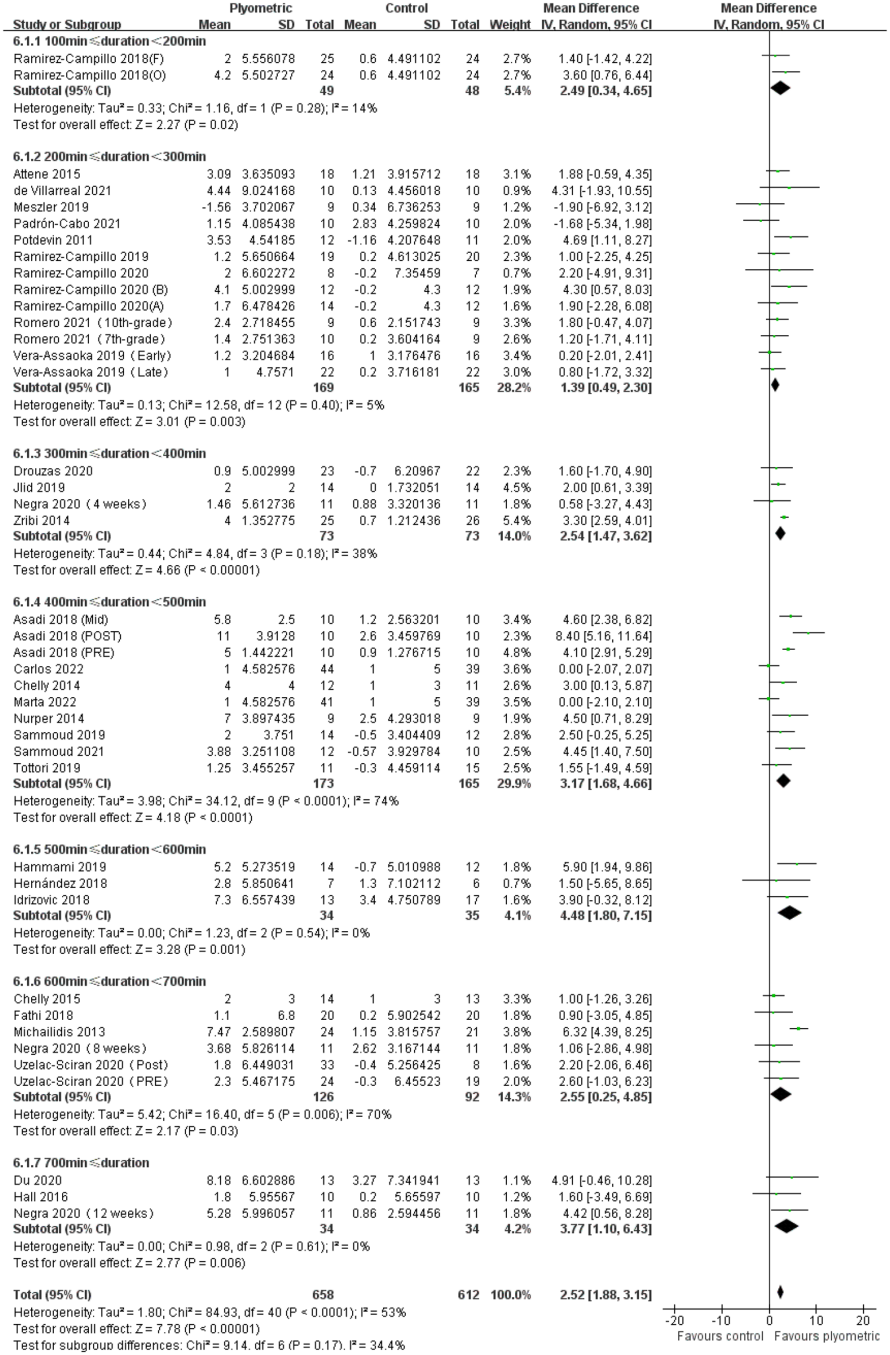
Forest plot of the effect of plyometric training with different overall intervention times on CMJ height in adolescents.

A total of 32 studies comprising 41 experimental groups and 1270 participants were included in this meta-analysis to evaluate the impact of plyometric training with different OITs on CMJ height in adolescents (Fig. 6). The results showed that plyometric training with different OITs had a positive effect on CMJ height in adolescents, including OIT 100–200 [MD = 2.49, 95% CI (0.34, 4.65), *P* = 0.02, I^2^ = 14%]; OIT 200–300 [MD = 1.39, 95% CI (0.49, 2.30), *P* < 0.01, I^2^ = 5%]; OIT 300–400 [MD = 2.54, 95% CI (1.47, 3.62), *P* < 0.01, I^2^ = 38%]; OIT 400–500 [MD = 3.17, 95% CI (1.68, 4.66), *P* < 0.01, I^2^ = 0%]; OIT 500–600 [MD = 4.48, 95% CI (1.80, 7.15), *P* < 0.01, I^2^ = 0%]; OIT 600–700 [MD = 2.55, 95% CI (0.25, 4.85), *P* = 0.03, I^2^ = 70%]; and OIT > 700 [MD = 3.77, 95% CI (1.10, 6.43), *P* < 0.01, I^2^ = 53%].

**Figure 7:**
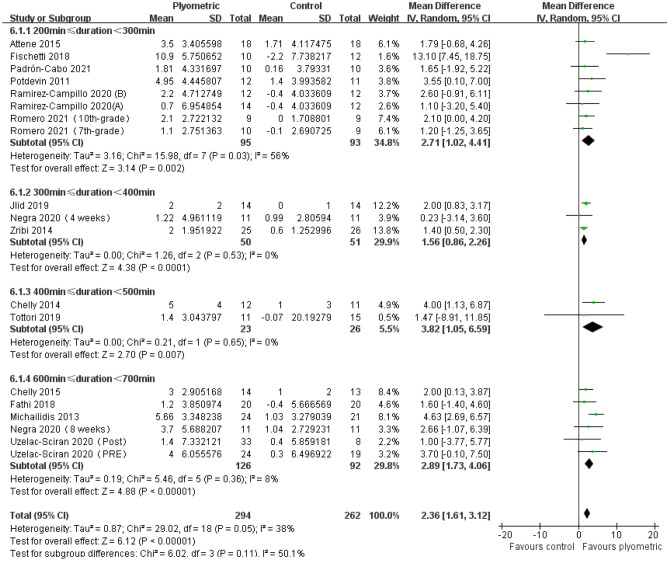
Forest plot of the effect of plyometric training with different overall intervention times on SJ height in adolescents.

A total of 15 studies comprising 19 experimental groups and 556 participants were included in this meta-analysis to evaluate the impact of plyometric training with different OITs on SJ height in adolescents (Fig. 7). The results showed that plyometric training with different OITs had a positive effect on SJ height in adolescents, including OIT 200–300 [MD = 2.71, 95% CI (1.02, 4.41), *P* < 0.01, I^2^ = 56%]; OIT 300–400 [MD = 1.56, 95% CI (0.86, 2.26), *P* < 0.01, I^2^ = 0%]; OIT 400–500 [MD = 3.82, 95% CI (1.05, 6.59), *P* < 0.01, I^2^ = 0%]; OIT 600–700 [MD = 2.89, 95% CI (1.73, 4.06), *P* < 0.01, I^2^ = 8%].

### Reporting bias

**Figure 8:**
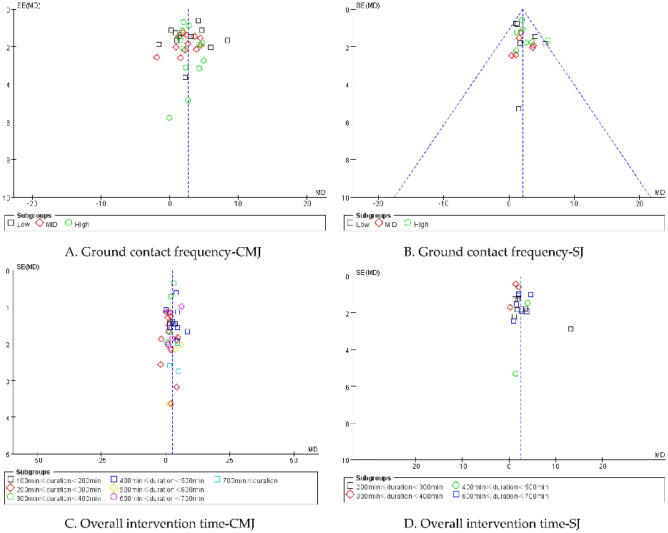
Plot of publication bias.

The symmetrical distribution in the funnel plots did not suggest the presence of publication bias (Fig. 8).

## Discussion

### The relationship between TGCF and jumping performance

The results of the meta-analysis indicate that plyometric training is an effective method for improving jumping performance, which supports the view that plyometric training can improve lower limb explosiveness^8,24,25^. Subgroup analyses indicate that different TGCTs induce specific adaptations in each type of jump. For the CMJ, it appears that LGCF is more beneficial, followed by MGCF and HGCF. In the case of SJ, HGCF seems to yield better results, followed by MGCF and LGCF. Similar to the results of most meta-analyses on the effects of plyometric training on jumping ability^8,9,25,26^, this study also found that plyometric training is an effective method for improving jumping performance in adolescents. Mark et al.^27^ conducted a similar experimental study to this one, where they divided all participants into low, moderate, and high-intensity training groups based on the different ground contact frequencies per week. Their results showed that low-intensity training can achieve similar effects as moderate and high-intensity training in the reactive strength index (RSI) measure. In contrast to Mark et al.^27^, this study classified the TGCF of the intervention, rather than just the frequency per week, and used CMJ and SJ as outcome measures to investigate the effects of different ground contact frequencies on jumping ability.

The primary reason for the improvement in jumping ability following plyometric training is the enhancement of central nervous system adaptation and muscle strength and explosiveness. Specifically, neural adaptation is characterized by changes in muscle activation strategy, which involve increased activation of agonist muscles and decreased activation of antagonist muscles^28^, or improvements in muscle excitability resulting from lengthening and shortening^29^. The improvement in muscle strength and explosiveness is mainly due to changes in muscle structure, such as a reduction in muscle fascicle angle and an increase in muscle fascicle length^30,31^, and changes in the stiffness of various elastic components, such as the plantar flexor-tendon complex^32–35^. These neurophysiological changes can enhance the efficiency of energy storage and release during the stretch–shortening cycle (SSC), ultimately resulting in improved jump performance^36^.

However, for the results of this meta-analysis, it appears that LGCF can better promote the improvement of CMJ ability in adolescents. Currently, there is not enough evidence to explain this phenomenon, and the physiological mechanisms underlying the effects of plyometric training with different TGCF on CMJ ability are not yet clear. Therefore, the following summarizes the potential mechanisms by which plyometric training with LGCF can better induce improvements in CMJ ability in adolescents:

1. CMJ is a widely used method for testing lower limb muscle strength and explosiveness, characterized by utilizing the SSC effect during jumping to generate higher jump heights^37^. The SSC effect not only involves the intrinsic biomechanical mechanisms of muscles but is also closely related to adaptive regulation of the nervous system^38^. In the SSC process, the muscle can activate more motor units for rapid contraction after being rapidly stretched, requiring the neuromuscular system to respond sensitively and accurately to signals of muscle lengthening^39^. LGCF may be more conducive to inducing neural adaptation, thereby further enhancing the SSC effect^38^

- Adolescents are at a peak period of growth and development, which makes them more susceptible to over-fatigue and sports injuries^40^. Compared to adults, adolescents have a greater neural load, making it difficult to effectively regulate the SSC process^41^. Therefore, plyometric training with LGCF can effectively reduce the neural load, promote neural monitoring and control of SSC^42,43^. Reducing training intensity appropriately can reduce neural fatigue, allowing for greater attention to SSC regulation, thus enhancing neural adaptation^44,45^.

(3) During high-intensity rapid muscle contractions, a large amount of lactate is produced, leading to muscle soreness and fatigue^46^. The accumulation of lactate in the muscle can inhibit muscle contraction ability, reducing muscle strength and explosiveness^47^. In addition, rapid muscle contraction requires a significant amount of energy, including ATP, creatine phosphate, and glycogen, among others. Energy depletion is also an important factor contributing to muscle fatigue^47–50^. Therefore, LGCF can reduce muscle lactate accumulation and fatigue, helping athletes maintain stable jumping performance^51–53^.

In contrast to the CMJ indicator, plyometric training with HGCF can better induce improvements in SJ ability. SJ is also a test method for measuring lower limb muscle strength and explosiveness, characterized by involving only concentric force during jumping^54,55^. More TGCFs in plyometric training can better induce improvements in maximal strength and explosiveness, thereby improving adolescent SJ ability. The following are the related reasons for this phenomenon:

1. When muscles are stimulated by nerve impulses, the release and reuptake of intracellular calcium ions (Ca2 +) are important regulatory mechanisms for muscle contraction and relaxation^56^. After extensive training involving repeated contractions and relaxations, the muscle's ability to release and reuptake Ca2 + can be improved. This, in turn, enhances the generation of muscle tension and relaxation speed, and helps the muscle adapt to higher loads and stronger stimuli, thereby enhancing muscle strength and endurance^50,57–59^.
2. With an increase in training frequency, motor neurons will connect to appropriate muscle fibers more frequently and make the synthesis and release of neurotransmitters more efficient^50,60–62^. This leads to clearer transmission of nerve impulses to the muscles, thus activating more muscle fibers^63^.

### The relationship between OIT and jumping performance

The results of this study indicate that the OIT for plyometric training is more reasonable in the range of 400–600 min for improving the jumping ability of adolescents. Subgroup analysis shows that for the CMJ index, the OIT for plyometric training can achieve the best training effect in the range of 500–600 min. As for the SJ index, the training effect is better with a OIT of 400–500 min.

Despite many meta-analyses on the effect of plyometric training on the jumping ability of adolescents, there has been no study summarizing time recommendations for plyometric training in adolescents^8,9,25,26^. Therefore, there is currently no clear recommendation for the most suitable OIT for plyometric training in adolescents. Negra et al.^64^ conducted a similar experimental study to explore the time effect of plyometric training on lower limb explosive power in adolescents with different total training weeks. The research team conducted four tests on the plyometric training group at baseline, week 4, week 8, and week 12. The results showed that the jumping ability of the plyometric training group improved significantly at week 12. Although Negra et al.^64^ study compared the training differences of plyometric training with different training weeks, the OIT, i.e. the training time per week and the OIT, was not further refined due to the long time intervals between weeks 4, 8, and 12. Currently, there is no study that categorizes plyometric training with different OITs into levels, so it is unclear how long plyometric training should be considered a short or long OIT for adolescents. Therefore, to further explore the effect of plyometric training with different OITs on the jumping ability of adolescents, this study further subdivided the OIT into subgroups of 100 min each, in order to more accurately investigate the potential relationship between OIT and the jumping ability of adolescents, and to provide more specific training recommendations for actual training.

OIT is one of the important factors affecting the effect of plyometric training. Too long OIT may lead to athlete fatigue, overtraining, sports injuries, and even a decrease in training effect^65,66^. Conversely, too short OIT may result in poor training effect, failure to fully stimulate athlete potential, and limit the development of athletic performance^67^. Therefore, a reasonable OIT is crucial for optimizing the training effect, especially for adolescents. The importance of a reasonable OIT is reflected in the following aspects:

1. A reasonable OIT for plyometric training can provide adolescents with sufficient training volume, which is crucial for the development of the neural and muscular systems and for improving jumping performance. Studies have shown that appropriate training volume can gradually help adolescents adapt to training, enhance the reaction speed and coordination of the neural-muscular system^44,68^. In terms of the neural system, appropriate training can improve the activation efficiency of motor units and the learning efficiency of movement patterns^69^. In terms of the muscular system, appropriate training can enhance muscle strength, explosive power, and elasticity, and improve the coordination between the neural and muscular systems^44,68^. These changes and improvements in the neural and muscular systems can enhance the neural-muscular reaction speed and coordination of adolescents, thereby improving their jumping ability^70,71^
2. Plyometric training positively impacts adolescent growth and development. Training duration and intensity play a crucial role in shaping the body and physiological systems^72,73^. During adolescence, growth hormone levels increase, stimulating the secretion of insulin-like growth factor 1 (IGF-1). This hormone promotes the growth of bones, muscles, and ligaments^74,75^. Plyometric training aligns with the body's heightened growth hormone secretion, enhancing explosive power and muscle strength. With appropriate duration, it further stimulates IGF-1 secretion, fostering the healthy development of bones, muscles, and ligaments. This helps adolescents improve bone density and reduce fracture risks^76–78^.

## Limitations and future research lines

### Limitations

Based on extensive experimental research on plyometric training for adolescents, this study conducted an in-depth statistical analysis of TGCF and OIT involved in plyometric training. However, we must acknowledge that due to the uniqueness of each experiment, we were unable to effectively correlate TGCF and OIT with intervention periods. Additionally, we recognize that the types of training movements have an important impact on training intensity and effects. However, in this study, we failed to quantify the intensity of various training movements involved in plyometric training, which may have affected our comprehensive understanding of the training effects.

### Future research lines

Moving forward, we will strive to collect more experimental research related to adolescent plyometric training, particularly studies that closely associate TGCF and OIT, as well as movement intensity, with intervention periods. We hope that through this approach, we can provide more accurate and comprehensive data and conclusions to inform both the theory and practice of plyometric training.

## Conclusion and practical applications

In conclusion, plyometric training has been proven effective for enhancing the jumping ability of adolescents. This meta-analysis highlights the importance of two key variables, namely TGCF and OIT, in influencing training outcomes.

The findings suggest that plyometric training with a LGCF (less than 900 contacts) is beneficial for improving CMJ ability in adolescents. On the other hand, plyometric training with a HGCF (more than 1400 contacts) is more effective for enhancing SJ ability. Additionally, the optimal OIT for improving jumping ability through plyometric training is within the range of 400–600 min. Specifically, an OIT of 500–600 min yields the best results for CMJ ability, while a training time of 400–500 min is more ideal for SJ performance.

Based on these findings, the following practical applications can be recommended:

1. Plyometric training is highly recommended for jump training in adolescents due to its effectiveness in enhancing their jumping ability
2. The determination of TGCF should be based on the training objectives. To improve CMJ ability, it is recommended to limit the TGCF below 900. For those aiming to improve SJ ability, a TGCF exceeding 1400 can be selected
3. OIT is a significant factor that affects training outcomes. For optimal improvement in CMJ ability, a total training time of 500–600 min is recommended. On the other hand, for better SJ performance, a training time of 400–500 min is more suitable.

## Data Availability

The datasets used and/or analysd during the current study are available from the corresponding author on reasonable request.

## Abbreviations

SSC: Stretch–shortening cycle
CMJ: Countermovement jump
SJ: Squat jump
RCTs: Randomized controlled trial studies
PRISMA: Preferred reporting items for systematic reviews and meta-analyses
WMD: Weighted mean difference
PT: Plyometric training
CON: Control group
PE: Physical education
PPT: Progressed plyometric training
NPPT: Non-progressed plyometric training
Ca2 +: Calcium ions
TGCF: Total Ground Contact Frequency
OIT: Overall Intervention Time
IGF-1: Insulin-like growth factor 1
LGCF: Low ground contact frequency
MGCF: Medium ground contact frequency
HGCF: High ground contact frequency
